# Sandwich-structure transferable free-form OLEDs for wearable and disposable skin wound photomedicine

**DOI:** 10.1038/s41377-019-0221-3

**Published:** 2019-12-09

**Authors:** Yongmin Jeon, Hye-Ryung Choi, Jeong Hyun Kwon, Seungyeop Choi, Kyung Mi Nam, Kyoung-Chan Park, Kyung Cheol Choi

**Affiliations:** 1 School of Electrical Engineering, Korea Advanced Institute of Science and Technology (KAIST), Daejeon, 34141 Republic of Korea; 2 Department of Dermatology, Seoul National University Bundang Hospital (SNUBH), Seongnam, 13620 Republic of Korea

**Keywords:** Optoelectronic devices and components, Organic LEDs

## Abstract

Free-form optoelectronic devices can provide hyper-connectivity over space and time. However, most conformable optoelectronic devices can only be fabricated on flat polymeric materials using low-temperature processes, limiting their application and forms. This paper presents free-form optoelectronic devices that are not dependent on the shape or material. For medical applications, the transferable OLED (10 μm) is formed in a sandwich structure with an ultra-thin transferable barrier (4.8 μm). The results showed that the fabricated sandwich-structure transferable OLED (STOLED) exhibit the same high-efficiency performance on cylindrical-shaped materials and on materials such as textile and paper. Because the neutral axis is freely adjustable using the sandwich structure, the textile-based OLED achieved both folding reliability and washing reliability, as well as a long operating life (>150 h). When keratinocytes were irradiated with red STOLED light, cell proliferation and cell migration increased by 26 and 32%, respectively. In the skin equivalent model, the epidermis thickness was increased by 39%; additionally, in organ culture, not only was the skin area increased by 14%, but also, re-epithelialization was highly induced. Based on the results, the STOLED is expected to be applicable in various wearable and disposable photomedical devices.

## Optoelectronics: New device lights the way for wearable healing patches

Korean scientists have developed an ultrathin flexible optoelectronic device for use in patches that can be worn on the skin to promote wound healing or to treat diseased cells. Optoelectronic devices that have no fixed shape and are flexible, wearable and disposable are attracting considerable attention as they can be placed close to the skin, providing an effective way to treat wounds. However, making conventional wearable optoelectronics requires complicated processes and produces poorly performing devices with short operating lifetimes. Now, Kyung Cheol Choi and colleagues from the Korea Advanced Institute of Science and Technology and Seoul National University Bundang Hospital in the Republic of Korea have made a reliable and high performing sandwich-structure ultrathin organic light-emitting diode. The device paves the way for wearable and disposable patches for healing wounds and other types of phototherapy.

## Introduction

With the advent of the 4th industrial revolution, it has become increasingly important to enhance the hyper-connectivity between devices and people. Devices that have no fixed shape and are flexible, stretchable, wearable, and disposable are being developed for such applications^1–10^. Among them are certain types of free-form optoelectronic devices, which can be attached close to the human body based on their conformal design. This conformal design provides a convenient and effective platform for performing a variety of potential functions, such as a sensor function for receiving information^11–13^, a display function for displaying information^11,14–17^, an energy production function, and a photomedicine function for managing health^18–21^. It is expected that multiple free-form optoelectronic devices, in close contact with the human body, will be conveniently and simultaneously used for various functions.

Most conventional conformable optoelectronic devices have been based on the use of organic light-emitting diodes (OLEDs) or quantum dot light-emitting diodes (QLEDs), which can be fabricated using low-temperature processes on flat thin film polymers^11,14,22,23^. However, these thin film-based optoelectronic devices have been limited to certain materials and shapes because their fabricated surface roughness is normally a few nanometres. Various approaches have been studied to fabricate unusual flexible optoelectronic devices without such limitations. Materials with high roughness, such as paper^24–26^ and textiles^27–29^, have been fabricated using synthesis or planarization processes. Recently, 3D-shaped optoelectronic devices^30–32^ have been developed using a new process.

In addition, methods of manufacturing optoelectronic devices on an ultra-thin substrate and then laminating them onto skin or other substrates have been reported^11,14,33,34^. Using these methods, it was possible to fabricate optoelectronic devices with various materials and shapes. However, these approaches have limitations, such as complicated processing methods, handling problems because the films are very thin, relatively low device performance, and short operating lifetimes.

Among the various potential uses of optoelectronic devices with conformable features, it is thought that photomedical applications are particularly promising. Such photomedical applications could include devices that promote biochemical reactions in the human body (photobiomodulation) or that selectively destroy diseased cells (photodynamic therapy) using an OLED light source^21,35–38^. Such conformable OLEDs can be more effective in terms of therapeutic effects because they can efficiently transmit light to the skin since they are located close to the human body.

Most photomedical research has been conducted using rigid LED^39,40^, rigid OLED^36–38^, and rigid QLED^41^ light sources. Recently, however, research has been conducted on patches for wound healing that utilize the advantages of conformable OLEDs^21,35^, and photomedical studies have also been conducted using conformable QLEDs^42^. Because conformable OLEDs have a surface light source, they can be attached to the human body, which provides several advantages, including low heat generation, light uniformity, and being ultra-thin and light weight^21^. However, to achieve improved photomedical effects and more convenient applications, research needs to be conducted to develop optoelectronic devices with a wider variety of shapes and materials.

In this study, we report a 10-μm-thick, ultra-thin, sandwich-structure transferable OLED (STOLED) that will allow optoelectronic devices to be freely formed on substrates of any material or shape. With this STOLED, free-form optoelectronic devices can be fabricated on materials such as textiles, skin, and paper, which are not suitable for conventional organic optoelectronic devices. Devices can also be fabricated on 3D-shaped cylinders and large-area arrays. In addition, the free-form OLEDs fabricated in this way can exhibit high efficiency (79.4 cd/A), equivalent to fabrication on glass. The ultra-thin transferable barriers (4.8 μm) sandwiching the OLEDs consist of 2 dyads of ZnO, Al_2_O_3_, MgO (ZAM) laminated film and a SiO_2_-based polymer. They provide high barrier performance (6 × 10^−6^ g/m^2^/day) and transparency (>86%) even in air or water environments. The STOLED can be fabricated on textiles because of its ultra-thin film characteristics and sandwich structure, and the neutral axis of the OLED can be freely adjusted. The reliability of the STOLED has been demonstrated, and it can withstand 1000 folding cycles. In addition, the STOLED retains its reliability after washing and exhibits a long operating life of more than 150 h. We examined the application of a red STOLED to keratinocytes, artificial skin models, and rat skin to determine its applicability to real wearable photomedical uses.

## Results

### Design and manufacture of the STOLED

**Fig. 1:**
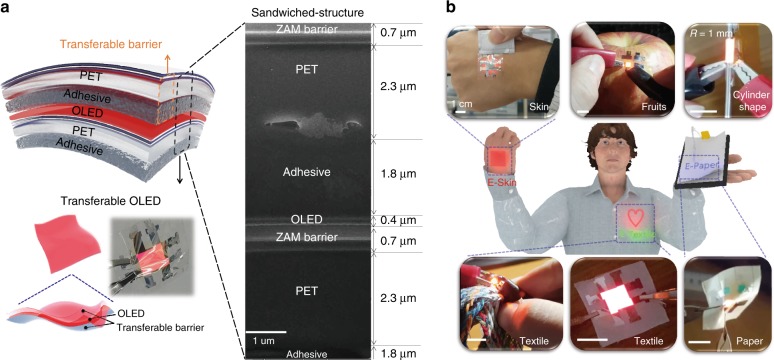
Demonstration of free-form OLEDs using the STOLED: **a** STOLED structure and FIB-SEM cross-sectional image. **b** Photo of various free-form OLEDs using STOLEDs with various materials and shapes.

The STOLED was fabricated by sandwiching an OLED within transferable barriers, as shown in Fig. 1a. The transferable barriers consist of a 2.3-μm-thick PET film on a 1.8-μm-thick acrylic adhesive (1100 MAS#05, SOOKWANG TTI Co. Ltd) and a 0.7-μm-thick encapsulation layer. The encapsulation layer consists of 2 dyads of ZnO, Al_2_O_3_, MgO (ZAM) film and a Si-based polymer. The combination of the OLED of ~0.4 μm sandwiched between transferable barriers of 4.8 μm leads to a total STOLED device thickness of only 10 μm.

When conventional OLEDs are directly fabricated on textile or paper using the standard manufacturing method (Fig. S1a), because of the high roughness of the substrate, the OLED device is typically short-circuited or electrically open, as shown in Fig. S1b. To solve this problem, various specific approaches have been developed for manufacturing OLEDs on unusual flexible substrates^24–32^. For example, for textile-based OLEDs, a thick planarization process with thermal lamination is required^27–29^, and only certain heat-resistant textiles that can withstand the thermal ALD used for encapsulation can be employed. Paper-based OLEDs have been reported that can only be fabricated on synthetic paper substrates with smooth surfaces^24–26^. Cylindrical-shaped OLEDs can only be fabricated by certain processes, such as dip-coating^30–32^. In other words, separate, different processing methods are required, depending on the material and shape of the substrate.

In contrast, the STOLED developed in this study provides a reliable free-form OLED for materials that degrade at high temperature, such as skin and fruit, as well as paper or textiles and other rough substrates, as shown in Fig. 1b. In addition, OLEDs are generally fabricated in planar form because of their processing methods, such as deposition and spin coating processes. However, the free-form OLED in the present study can also be used to fabricate 3D-shaped OLEDs in the form of cylinders (Fig. 1b). The present STOLED can be fabricated as a free-form OLED independent of the material and shape using the same method. These free-form OLEDs can be employed in various types of optoelectronic devices, including wearable and disposable displays using E-Skin, E-Textile, and E-paper, and through their use, photomedical health care applications can be realized. Previously, we reported that a red-wavelength OLED may have excellent effects in wound healing through fibroblast proliferation and migration effects^21^. In this study, the effects of the STOLED on keratinocytes were tested using cultured keratinocytes and skin equivalent models to examine the effects of the STOLED on skin composed of epidermis and dermis.

**Fig. 2:**
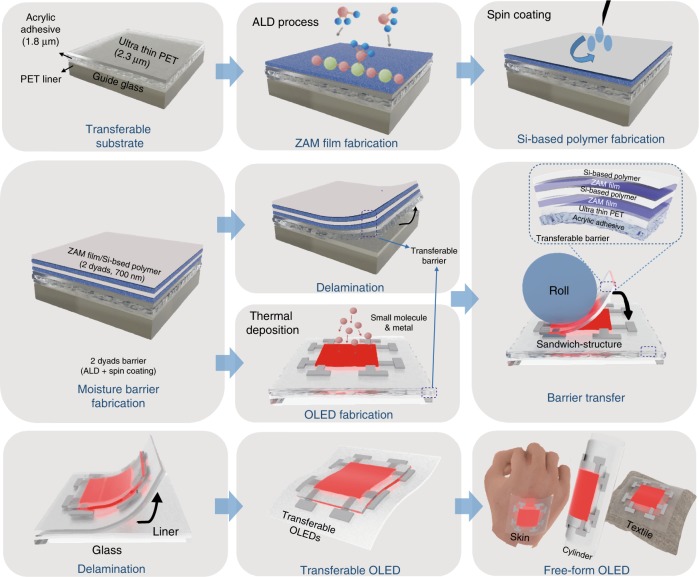
STOLED fabrication; process of sandwiching an OLED within transferable barriers.

The STOLED was fabricated in the order shown in Fig. 2. First, a 50-μm-thick PET release liner, which will be removed before transfer, is fixed onto the glass guide. A 1.8-μm-thick acrylic adhesive is formed on the PET release liner, on which a 2.3-μm-thick ultra-thin PET layer is formed to make a transferable substrate. Second, a process is carried out to fabricate a barrier over the transferable substrate so that the OLED can be reliably operated in air and moisture environments. This process results in encapsulation by the 2-dyad structure (ZAM/polymer/ZAM/polymer) and involves fabricating the ZAM film using atomic layer deposition (ALD) and fabricating the Si-based polymer using a spin coating process^43^. After the transferable barrier is formed, a portion of the barrier is used to fabricate the OLED through direct thermal deposition on top. The remainder is delaminated from the PET release liner and transferred to the OLED to provide a barrier function. Similar to transferable barriers, STOLEDs can be separated from the PET release liner and freely transferred regardless of the material or shape to form free-form OLEDs. Various free-form OLEDs fabricated in this way were reliably operated, as demonstrated in Supplementary Video S1–S3.

### Structure and performance of the transferable barrier

In the conventional method of fabricating unusual OLEDs, the ALD process is directly performed on the device, and a thin film with an excellent barrier function can be formed. However, the process speed is very slow, and the device may become unstable due to heat damage (Fig. S2a). Additionally, because of their process temperatures, most of the previously reported unusual OLEDs can only be fabricated on heat-resistant substrates^27–29,44^. In addition, the encapsulation film sealing or lamination method requires the use of a resin or PDMS buffer cured by UV exposure or heating, and as a result, the device is thickened from several hundred microns to several millimetres, which severely limits the formation of free-form OLEDs^45,46^.

The transferable barrier proposed in this study is currently the only solution capable of forming a barrier regardless of the substrate material and shape (Fig. S2b). The transferable barrier introduced in this study does not require a curing process, and the ultra-thin (4.8 μm) barrier can be formed regardless of the shape and material of the substrate. By forming and sandwiching the OLED device within the barrier, it can remain ultra-thin, and a transfer process or special annealing is not required, which could damage the OLED device. In addition, the barrier is formed on the OLED very quickly using the R2R process, without the delay imposed by curing. The manufactured STOLED can be transferred to any substrate without additional planarization. Furthermore, a separate process is not required each time the OLED is fabricated on a different substrate, as in the conventional approach. As such, free-form OLEDs can be rapidly fabricated using the R2R process regardless of the type of flexible substrate.

**Fig. 3:**
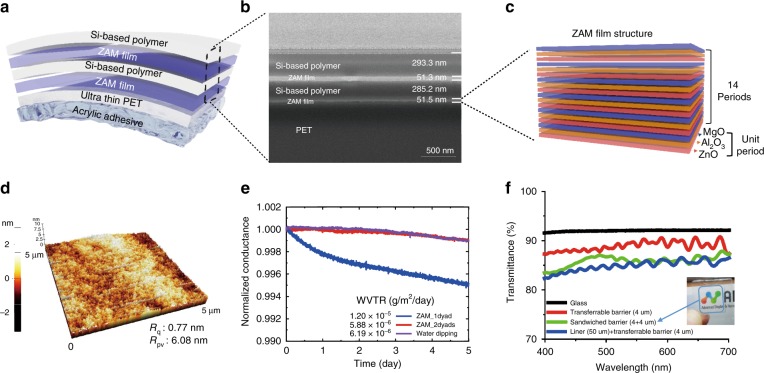
Structure and characteristics of the transferable barrier: **a** Structure of the free-standing transferable barrier. **b** FIB-SEM cross-sectional image of the barrier and the barrier thickness. **c** Laminated structure of the ZAM film composed of 14 unit layers consisting of ZnO, Al_2_O_3_, and MgO. **d** AFM images and surface roughness after barrier formation. **e** Ca test results for WVTR performance evaluation of the transferable barrier. **f** Transmittance characteristics of the transferable barrier.

The transferable barriers that enable simultaneous barrier and transfer functions have a 2-dyad structure of ZAM film and a Si-based polymer on ultra-thin PET containing an acrylic adhesive (Fig. 3a). The ZAM film is an oxide layer that protects the OLED from moisture and oxygen, and the Si-based polymer has a relatively low Young’s modulus of 1.8 GPa; thus, it is used to provide barrier flexibility and defect decoupling. As seen in the FIB-SEM image in Fig. 3b, the ZAM film and the Si-based polymer have thicknesses of 50 nm and 300 nm, respectively, in a transferable barrier.

As shown in Fig. 3c, the unit layer is formed with a 20/12/10 cycle ratio of ZnO, Al_2_O_3_ and MgO, and a total of 14 unit layer cycles is repeated to form the ZAM film with a thickness of 50 nm. The ZAM nanolaminate films form a quasi-perfect layer, which not only improves the flexibility by forming a crack arrestor in the oxide layer itself but also improves the barrier performance by performing a defect-decoupling function due to the numerous layers^43,47^.

The manufactured transferable barrier simultaneously planarizes the substrate and acts as a barrier. Because OLEDs are very thin, only several hundred nanometres can only be safely fabricated on substrates planarized at the few nanometre level. The bare transferable substrate shows a relatively high peak-to-valley roughness of 18.23 nm (Fig. S3a). After forming the 2-dyad barrier, it was planarized to a peak-to-valley roughness of 6.08 nm, as shown in Fig. 3d. After the barrier was fabricated, as shown in Fig. S3b, the uniformity of the OLED was greatly improved.

A Ca test was conducted to confirm that the transferable barrier function was applicable to an OLED device. As shown in Fig. 3e, when only 1 dyad was used, the water vapor transmission rate (WVTR) was confirmed to be 1.2 × 10^−5^ g/m^2^/day, which is relatively unsuitable for OLEDs. For the 2-dyad transferable barrier, the WVTR was 5.88 × 10^−6^ g/m^2^/day, and this barrier was determined to be the final barrier structure appropriate for OLEDs.

However, it is generally known that Al_2_O_3_, which constitutes the ZAM film, reacts with water to form a boehmite structure^35,48,49^. We used a SiO_2_-based polymer to prevent the ZAM film from reacting with water to form boehmite. An inactive silicon oxide (SiO_x_) capping layer was formed on the surface of the ZAM film because the silicidation process between the Si-based polymer and the ZAM film results in intense intermolecular condensation of Si-O-Si^35,48^. The inactive silicon oxide was able to prevent the reaction between Al_2_O_3_ and water and subsequently inhibit the formation of boehmite. The 2-dyad barrier was dipped in water for one hour, and a low WVTR (6.19 × 10^−6^ g/m^2^/day) was measured, even after the same Ca test. This barrier property means that a free-form OLED fabricated on clothes can be reliably operated not only in an air environment but also in an environment such as washing in water.

In addition, as shown in Fig. 3f, the transferable barrier fabricated on a 4-μm-thick substrate on a 50-μm-thick PET release liner showed 84.7% (λ = 550 nm) transmittance. The free-standing barrier, separated from the release liner to provide the actual barrier function, showed a very high transmittance of 89.0% (λ = 550 nm). However, in practical devices, the transferable barrier will be applied in a sandwich structure. Even when the free-standing barrier was sandwiched, when the transmittance was measured, a high transmittance of 86.0% (λ = 550 nm) was confirmed.

### Performance and reliability of the STOLED

**Fig. 4:**
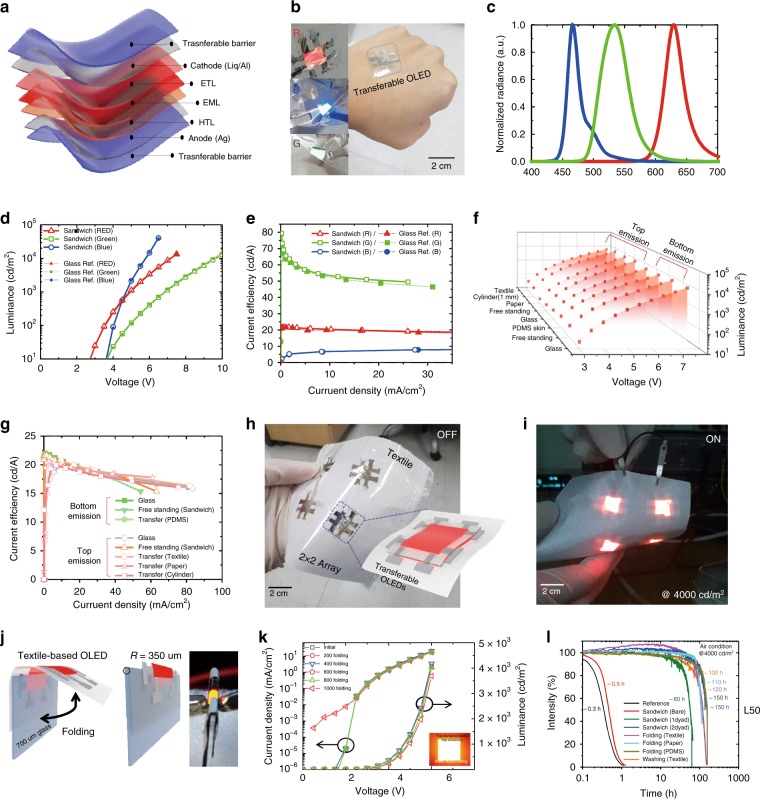
Characteristics and reliability of three-colour STOLEDs and free-form OLEDs: **a** Structure of the R/G/B STOLED. **b** Photo of an operating R/G/B STOLED and a free-form OLED transferred to skin. **c** Emission wavelength characteristics of the R/G/B STOLED. **d** Graph of the luminance/voltage of each R/G/B STOLED. **e** Graph of the current efficiency characteristics of the R/G/B STOLED. **f** Graph showing the same L/V characteristics of red free-form OLEDs fabricated with various materials and shapes. **g** Graph showing the same current efficiency characteristics of red free-form OLEDs fabricated with various materials and shapes. **h** Photo of a large-area red free-form OLED with a 2 × 2 array on textile. **i** Large-area red free-form OLED driven by a 2 × 2 array. **j** Schematic image of the folding test and photo of an operating OLED while folded. **k** Driving characteristics of an OLED in relation to folding cycle (0–1000 cycles). **l** Lifetime graph of a free-form OLED.

Three-colour STOLEDs, made by sandwiching OLEDs between the transferable barrier, were constructed, as shown in Fig. 4a. Three-colour STOLEDs are commonly fabricated between an anode made of 30-nm-thick silver and a cathode made of 1-nm-thick 8-quinolinolato lithium (Liq) and 100-nm-thick aluminium. The R/G/B OLEDs fabricated between the electrodes are described in more detail in the experimental section. The three-colour STOLED fabricated in this way not only is very flexible, as shown in Fig. 4b, but also can be freely fabricated on rough surfaces such as skin.

The three-colour STOLEDs have emission wavelengths of λ_peak_ = 629 nm for red, λ_peak_ = 534 nm for green, and λ_peak_ = 466 nm for blue (Fig. 4c, Table 1). All three-colour STOLEDs were capable of being driven at a low voltage, within 10 V, with a high efficiency of 21.6 cd/A for red, 79.4 cd/A for green and 8.3 cd/A for blue (Fig. 4d, e, Table 1). Previously reported unusual OLEDs have not been able to match the performance of glass-based OLEDs due to limitations in their manufacturing method^27,31^. However, the STOLEDs fabricated in the present study have the same electrical and optical performance as OLEDs fabricated on glass substrates (Fig. 4d, e). Therefore, photomedicine using STOLEDs should have the same high therapeutic effect as that using conventional OLEDs and can be applied in new photomedical applications, such as wearable, implantable, and attachable devices, based on additional advantages such as the form factor. These characteristics are superior to those of the reported textile-based OLEDs^27–29^ and paper-based OLEDs^24–26^. Even when free-form OLEDs were fabricated by transferring a red STOLED to textile, paper, PDMS, glass, etc., the electric and optical characteristics of the devices were almost the same (Fig. 4f, g). The free-form OLED can be manufactured regardless of the type of substrate. The STOLEDs can be applied to a large-area free-form OLED because they can be fabricated in an array (2 × 2) on textile, as shown in Fig. 4h. Free-form OLEDs with a 2 × 2 array fabricated in series on a large-area textile operated normally, as shown in Fig. 4i.

**Table 1:** Three-colour STOLED electrical and optical characteristics.

Index colour	λ_peak_ [nm]	FWHM [nm]	η_max_ [cd/A]	η_max v_ [V]	L_max_ [cd/m^2^]	Voltage [V] @1000 cd/m^2^
Red	629	34	21.6	4	13,040	4.8
Green	534	51	79.4	5	12,858	6.9
Blue	466	22	8.3	5.5	40,525	4.7

To confirm the reliability of the STOLED, textile-based OLEDs were fabricated by transferring the STOLED onto 100-μm-thick 100% polyester textile. The Peirce cantilever test was conducted to confirm that the original flexible properties of the textile remained even after the transfer of the STOLED^50,51^. As shown in Fig. S4, the cantilever length of the bare textile, 2.3 cm, indicated that the textile was very flexible, and the cantilever length of the free-standing STOLED was 3 cm, similar to that of the bare textile. Even after transferring the STOLED to the textile, the cantilever length was 3.8 cm, confirming that the flexibility of the textile was not significantly lost. The cantilever length characteristics of the textile-based OLED fabricated using a very simple transfer method in this study were similar to those fabricated by conventional complex methods^27–29^. In addition, the cantilever length was maintained at a similar level for 40-μm-thick PDMS (2.6–3.9 cm) and 30-μm-thick paper (11.1–13.4 cm) onto which the STOLED was transferred (Fig. S4). That is, the STOLED could be transferred while maintaining the flexibility regardless of the substrate.

The peeling force of the transferred textile-based OLED was measured using a peeling test to determine how well the textile was attached. As shown in Fig. S5a, the maximum peeling force was 65 N/m, so the OLED was not easily separated from the textile. Because of this peeling force, it was confirmed that the STOLED was not separated from the textile even when a washing test was carried out with a commercial washing machine. Additionally, the STOLED was well transferred regardless of the substrate, with a maximum peeling force of 65 N/m for paper and 50 N/m for PDMS (Fig. S5b, c). To confirm that the lighting performance of the STOLED is maintained even after peeling, a test was conducted on the textile with the largest average peeling force. Even after three transfers and peelings, as shown in Fig. S6, the lighting performance of the STOLED was maintained.

To test the flexibility of the textile-based OLED, a folding test was carried out using the method shown in Fig. 4j. In the folding test, one end of the textile-based OLED was fixed on 700-μm-thick glass to permit a folding radius of 350 μm. As shown in Fig. 4k, when the devices were folded 1000 times, the device characteristics were almost the same as those before folding (Supplementary Video S4). This is a higher flexibility than that found for any previously reported textile-based OLED^27–29^. This folding stability was maintained not only for the textile but also for the STOLEDs transferred to PDMS or paper substrates (Fig. S7).

The reason for this flexibility is that the OLEDs are fabricated with a sandwich structure between ultra-thin PET films. As the transferable substrate becomes thinner, the strain decreases, and the neutral axis is closer to the OLED device. However, the neutral axis remains below the OLED, regardless of how thin the substrate is (Fig. S8a, c), unless an extra thick barrier is formed on the OLED. In this case, if the OLED is transferred to a textile, the neutral axis moves farther from the OLED (Fig. S8a).

However, the STOLED forms a neutral axis above the OLED because the OLED is sandwiched within the barriers. This is because the relatively high Young’s modulus, 150 GPa, of the ZAM films is not perfectly symmetric about the OLED but is concentrated at the top of the PET. The neutral axis being formed on the OLED after it is transferred to another substrate is advantageous.

As shown in Fig. S8b, when the STOLED is transferred to a 100-μm-thick textile that has a low Young’s modulus of 0.6 MPa, the neutral axis is located at the centre of the OLED device. Unlike the reference device, where the neutral axis moved away from the OLED after transferring it to the textile, as shown in the graph in Figure S8c, the STOLED neutral axis is accurately located in the OLED. This method provides the advantages of flexible, ultra-thin OLEDs while simultaneously adjusting the neutral axis location so that the STOLED remains highly flexible after transfer. This means that the STOLED can be applied to substrates with other Young’s moduli, and the neutral axis can be located at the centre of the OLED by adjusting the thickness ratio of the acrylic adhesive or PET in the sandwich structure.

Textile-transferred STOLEDs were tested for washability and reliability after exposure to water. As shown in Fig. 3e, the Si-based polymer prevented boehmite formation and provided a reliable barrier in water^48^. Because of these characteristics, the STOLED was confirmed to have the same performance even after 20 cycles of stirring for 15 min at 200 rpm in water (Fig. S9). The STOLED was also determined to have an operating reliability of 150 h or more, as shown in Fig. 4l, unlike conventional ultra-thin film-based OLEDs, which have a short operating life of up to 2–30 h^14^. It has also been confirmed that the STOLED has a long operating life of more than 100 h even after being folded 1000 times and stirred in water.

In this study, we were able to fabricate free-form OLEDs and improve both the performance and reliability compared to conventional unusual flexible optoelectronic devices (Table S1). The STOLED can be employed in various applications because it can be used to form an OLED anywhere with high reliability. As a result, by using STOLEDs, OLEDs can be placed close to the human body and applied in various wearable optoelectronic devices. Among various applications, wearable photomedical applications have many advantages if they can be realized close to the human body. As seen in Fig. S10, although the ratio of light received may vary depending on the size ratio of the light source and the detector (such as skin), it is clear that the amount of light reaching the detector sharply decreases when the light source is moved away from the detector. A wearable free-form OLED such as the STOLED can be conveniently applied for treatment, and the light reaching the skin can be maximized, thereby improving the therapeutic effect. To investigate this, we conducted an experiment to check whether the STOLED was applicable to photomedicine.

### Evaluating the effect of the STOLED for photomedical applications

**Fig. 5:**
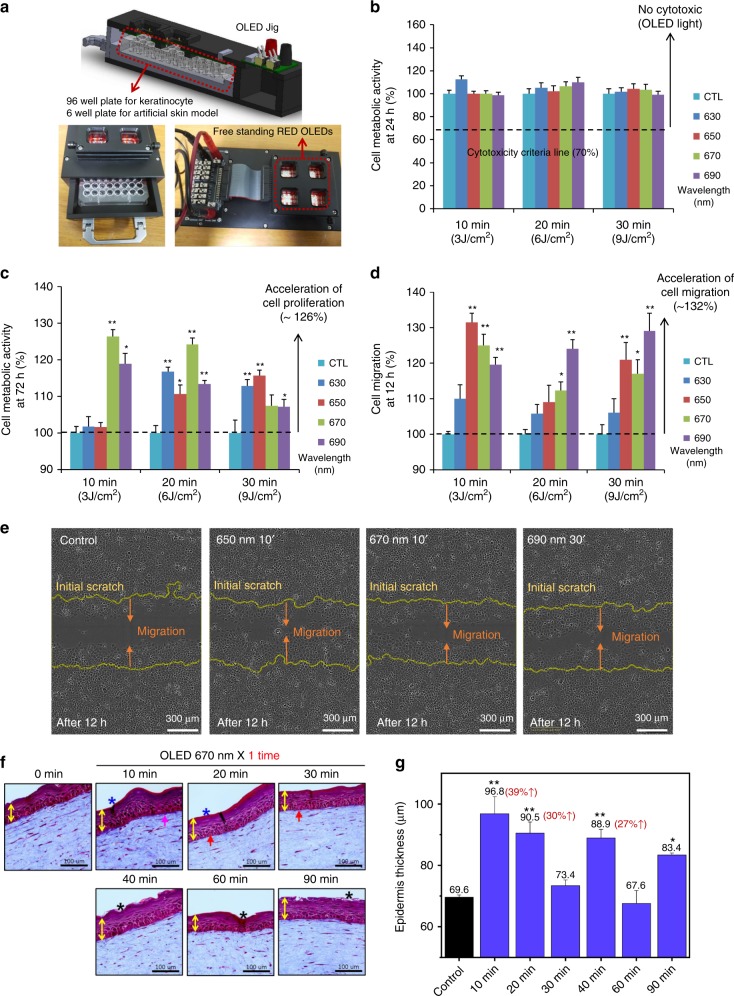
In vitro wound healing effect using keratinocytes in a 2D model and semi-in-vivo wound healing effects in a 3D artificial skin model using red STOLEDs. (The graphs are expressed as the mean ± mean error (*n* = 8), and all STOLEDs with peak wavelengths between 630 and 690 nm were used for irradiation with a power of 5 mW/cm^2^. Additionally, asterisk means that the p value is less than 0.05, and double asterisks mean that the p value is less than 0.01.): **a** OLED-driven jig design used for cell experiments. **b** Results of the keratinocyte cytotoxicity test 24 h after light irradiation. **c** Results of the keratinocyte cell proliferation test 72 h after light irradiation. **d** Results of the migration test 12 h after light irradiation. **e** Migrated cell images for 650 nm 10’, 670 nm 10’, and 690 nm 30’, which showed the best migration test results compared to the control group. **f** Photograph of the results using the artificial skin model when irradiated with a 670 nm STOLED for 0–90 min. **g** Graph of the thickness of the epidermis with time after STOLED irradiation using the artificial skin model.

We tested the performance of a flexible red-wavelength OLED in treating a wound by investigating the cell proliferation and cell migration effect using normal human fibroblasts based on previous studies^21^. In this study, experiments were extended to normal human keratinocytes, which are located at the outer surface of the skin. Studies were conducted using the OLED driving jig shown in Fig. 5a to determine whether a red-wavelength STOLED can be beneficial not only to fibroblasts but also to keratinocytes. Keratinocytes in 96-well plates were irradiated with the OLED, which was 15 mm from the keratinocytes.

Red-wavelength STOLED light with peak wavelengths of 630, 650, 670, and 690 nm (±5 nm) was irradiated for 10 (3 J/cm^2^), 20 (6 J/cm^2^), and 30 (9 J/cm^2^) min at an intensity of 5 mW/cm^2^ to study the cytotoxicity of irradiating keratinocytes. After STOLED irradiation, the cell metabolic activity was measured by the tetrazolium salt method via Cell Counting Kit-8 assays. As shown in Fig. 5b, when irradiated with a red-wavelength STOLED at a power of 5 mW/cm^2^ for up to 30 min, the cell viability was 100% or more. Therefore, it was confirmed that the STOLED was not cytotoxic to keratinocytes^52^. These results were the same as those obtained using red-wavelength OLED irradiation of fibroblasts^21^.

In addition to cytotoxicity testing, the metabolic activity of the keratinocytes irradiated with the STOLEDs was measured after 72 h to determine the effect on cell proliferation. With fibroblasts, the proliferative effects of red-wavelength OLEDs have been shown to vary with wavelength and energy^21^. Similarly, when the red-wavelength STOLEDs were applied to keratinocytes, the proliferation effect was different depending on the wavelength and energy (Fig. 5c). In particular, when keratinocytes were irradiated with the 670 nm STOLED for 10 min (3 J/cm^2^) to 20 min (6 J/cm^2^), the obtained proliferative effect was 26% (±5%) and 24% (±5%), respectively, compared to the control (*p* < 0.01).

Cell migration tests were also carried out using a scratch-wound healing assay. Because cell migration is also very important for effective wound healing, we observed cell migration after applying red-wavelength STOLEDs to keratinocytes (Fig. S11). In all STOLED irradiation conditions, the initial cell migration (<12 h) was accelerated, and scratch coverage was rapidly achieved after 18 h. As shown in Fig. 5d, e, 12 h after light irradiation, the three conditions of 650 nm for 10 min, 670 nm for 10 min (3 J/cm^2^), and 690 nm for 30 min (9 J/cm^2^) resulted in increases in cell migration of 32% (±3%), 25% (±3%), and 29% (±5%), respectively (*p* < 0.01).

In this study, the most efficient wavelength was selected based on the different biological responses. A wavelength of 630 nm is generally not effective for cell migration. A 10 min exposure to 630 nm or 650 nm light is not effective for cell proliferation, but a 650 nm wavelength is generally effective for cell migration. Ten minute and 20 min exposures to 670 nm light are very effective for cell proliferation, and this wavelength is generally effective for cell migration. Short-term exposure to 690 nm light is also effective for cell proliferation, and this wavelength is effective for cell migration. Thus, short exposure to short wavelengths, such as 630 nm and 650 nm, is not effective for cell proliferation. However, short exposure to longer wavelengths, such as 670 nm and 690 nm, is effective for cell proliferation. Additionally, the migration effects are different according to the wavelength and exposure duration. Clinically, it will be better if a shorter treatment is more effective than a longer treatment. Although there can be different situations and combinations of wavelengths, our results suggest that the 670 nm wavelength seems to be best in terms of immediate cell proliferation effects and cell migration effects.

Considering the effect on both proliferation and migration of different wavelengths and irradiated energy, it was concluded that irradiation at 670 nm for 10 min was the best condition for wound healing in terms of the keratinocyte response. Next, we tested the potential in skin equivalent models. The artificial human skin equivalent model, including both dermal and epidermal cells, can provide results to address physiological questions that cannot be answered with a monolayer cell culture^53^. Furthermore, it is considered a good in vitro model for observing the proliferative potential of keratinocytes and epidermalization because it allows the growth of keratinocytes that mimic the natural skin morphologically and biochemically and promote wound healing^54,55^.

To check the effect of the STOLED in the skin equivalent model, a STOLED with a 670 nm wavelength and an intensity of 5 mW/cm^2^ was used for irradiation from 10 min to 90 min. As shown in Fig. 5f, g, for 670 nm STOLED irradiation from 10 to 90 min, most of the epidermis became thicker than the control. Particularly, when the 670 nm STOLED was used for irradiation for 10 min and 20 min, the epidermal thickness greatly increased by 39% (±5%) and 30% (±3%), respectively. In addition, the histology of the epidermis was similar to that of normal skin in these conditions. In this set of studies, the effects of the 670 nm STOLED were not good when the skin equivalent model was irradiated for more than 30 min. This is because the photobiomodulation effect has the characteristics of a biphasic response^39^. This means that the therapeutic effect is not proportional to the irradiation time and irradiation energy; instead, after certain optimum conditions are reached, the effect is reduced. Furthermore, this tendency was observed for the cell proliferation and migration effect using cultured normal human keratinocytes, as shown in Fig. 5c, d. Rapid migration effects were observed at 10 min and 20 min with a 670 nm wavelength. In addition, collagen was increased with 10 min of irradiation, and the cell shape of the basal layer was improved at 20 min of irradiation. These findings suggest that short-term exposure to 670 nm light is well tolerated and can be used in wound healing situations.

To test the wound healing effects in an organ culture model, rat skin was used. First, a 6 mm hole was made in the centre of the skin fragments. After irradiation with the 670 nm STOLED for 10 min (Fig. S12a), skin fragments with a diameter of 16 mm were excised, and 1 ml of fibroblasts in collagen gel was poured into the hole. We assume that a skin fragment with an internal hole can act as a model to check the two directional proliferation and migration of cells. Organ cultures were conducted for 3 weeks (Fig. S12b). Interestingly, the total skin fragment size became larger in the irradiated model compared to the control group, as shown in Fig. S12c. These results suggest that 670 nm STOLED irradiation can induce cell proliferation and migration, accompanied by tissue growth.

**Fig. 6:**
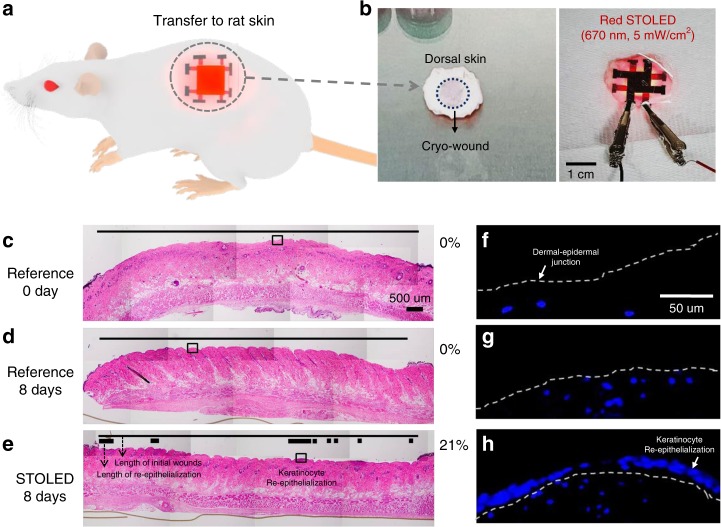
The effect of irradiating rat skin with a 670 nm STOLED on organ culture in the cryo-wound model (the thin lines (**c**–**e**) indicate the length of initial wounds, while the thick lines (**e**) indicate the length of re-epithelialization; the dotted lines (**f**–**h**) indicate the dermal-epidermal junction): **a** Conceptual image of a photomedical application, with a STOLED attached to rat skin. **b** Photograph of a STOLED attached to rat skin for the cryo-wound model. The photographs are **c**–**e** H&E- or **f**–**h** DAPI**-**stained paraffin sections. **c**, **f** A cryo-wound with all epidermal cells dead after 24 h of injury. The cut skins were **e**, **h** irradiated with a 670 nm STOLED or **d**, **g** not, followed by incubation for 8 days.

Next, the wound healing effect of the contact STOLED, which was not possible with conventional OLEDs, was examined using the rat skin cryo-wound model (Fig. 6a, b). Cryo-wounds with a diameter of 10 mm were made using liquid nitrogen, resulting in the cell death of the entire epidermal region after 24 h (Fig. 6c, f). The cut skin was irradiated with the 670 nm STOLED, followed by incubation for 8 days. Re-epithelialization was measured as the distance of the epidermis consisting of newly grown keratinocytes compared to the initial wound on a haematoxylin and eosin (H&E)-stained paraffin section.

In the irradiated wound (Fig. 6e), 21% re-epithelialization was clearly observed, but not in the non-irradiated control (Fig. 6d). Cell nuclear staining using DAPI also showed the same results (Fig. 6g, h). These results suggest that 670 nm STOLED irradiation may promote keratinocyte proliferation, migration, and re-epithelialization, leading to rapid and complete wound healing.

In summary, the STOLED increased the proliferation and migration of cultured normal human keratinocytes in a monolayer culture system. It also increased the thickness of the epidermis in the skin equivalent model. It also enhanced wound healing effects in organ culture models. Therefore, it can be concluded that the STOLED has beneficial effects on wound healing. These results still need to be evaluated in humans to confirm possible application in clinical practice and in various wearable or disposable photomedical devices.

## Discussion

In summary, a transferable sandwich-structure ultra-thin (10 μm) OLED was developed that is suitable for photomedical applications in wearable or disposable devices close to the human body. The barrier used to sandwich the OLED is composed of ZAM laminate film and a Si-based polymer, which inhibits boehmite formation, with a barrier performance of 5.88 × 10^−6^ g/m^2^/day, and can simultaneously planarize the substrate to the several nanometre level. At the same time, it exhibits a high transparency of 86% or more and can be readily transferred to various substrates.

Fabricated STOLEDs were freely transferred to skin, textile, paper, cylinders, etc. and could be manufactured with the same high performance (79.4 cd/A). Based on the structural characteristics of the STOLED, the neutral axis of the textile-based OLED can be accurately positioned at the centre of the OLED. The device performance was maintained even at a folding radius of 350 μm (for 1000 cycles). In addition, textile-based OLEDs retained the device performance even after 20 cycles of washing (15 min, 200 rpm) and were proven to have up to 150 h of operating reliability.

The complex process known as ‘wound healing’ consists of a series of cellular and molecular events, which are typically described as a sequence of three overlapping stages: inflammation, proliferation (or tissue formation), and tissue remodelling^56^. During the tissue formation phase, different parts of the damaged skin are repaired in the wound site. Re-epithelialization is the restoration of the damaged epidermis. Because mammalian epidermis comprises the surface of the skin and is a stratified epithelium made of keratinocytes, their proliferation and migration is essential for successful wound closure^57^.

In this study, the effects of different red-wavelength STOLEDs were investigated. Visible light is composed of different wavelengths from 400 nm to 700 nm. Among them, blue light is known to be antimicrobial. Recently, it was reported that blue light does not impair wound healing in vitro, but there is a concern that blue light may slow wound healing^58^. In addition, it is generally believed that red (660 nm) and near-infrared (810 nm) light stimulates cell proliferation, while short wavelength, such as blue (415 nm) and green (540 nm), light inhibits cell proliferation^59^. Recently, it was also reported that green light stimulates angiogenesis and myofibroblastic differentiation during the repair of third-degree skin burns. Red light may stimulate further re-epithelialization and wound retraction, especially in advanced repair phases^60^. Overall, the beneficial effects of red light in wound healing are well known compared to blue or green light. However, the effects of different red wavelengths have not been tested because of the limitations of the light sources, such as LEDs. Thus, we focused on the red wavelength and investigated the effects of different red wavelengths using OLEDs.

When keratinocytes were irradiated with red wavelength (630, 650, 670, and 690 nm) STOLEDs, there was no cytotoxicity, a 26% enhancement in cell proliferation and 32% accelerated cell migration. The increased proliferation and migration of epidermal keratinocytes are expected to accelerate and complete epidermal healing. In addition, when the STOLED was applied to the skin equivalent model, the epidermal thickness was improved by 39%. This result indicates that there was an increase in epidermal cell proliferation and subsequent promotion of re-epithelialization. When the STOLED was applied to the organ culture model using rat skin, not only was the skin area improved by 14%, but also, 21% re-epithelialization was definitely observed, induced by the proliferation of keratinocytes in a portion of the wound. These results show that the STOLED can affect wound healing by increasing the volume of tissue and promoting re-epithelialization in the damaged area.

This study has demonstrated the applicability of free-form OLEDs to wearable or disposable wound healing light therapy using a novel structural approach to realize high performance and high reliability. The method also allows the light source to be placed close to the human body, providing therapeutic advantages. Unlike a conventional rigid, point light source and non-contact-based wearable photomedical devices, the superior STOLED in this study can be employed in a wearable photomedical system with a conformable surface light source that can be attached to the skin^61^. The STOLED performance was demonstrated in a wearable and disposable photomedical device used in a wound healing test on human keratinocytes, in artificial skin models, and in animal models. Based on these observations, we can conclude that the STOLED is suitable not only for wound healing but also for various types of phototherapy close to the human body.

## Materials and methods

### OLED fabrication

The OLEDs were fabricated in a vacuum environment of 10^−6^ Torr using thermal evaporation equipment. R, G, B OLEDs are commonly fabricated with a silver electrode as an anode (30 nm), MoO_3_ as a hole injection layer (5 nm), an electron injection layer (1 nm), and an aluminium electrode as a cathode (100 nm). The red OLED has N,N′-di(1-naphthyl)-N,N′-diphenyl-(1,1′-biphenyl)-4,4′-diamine (NPB) as a hole transport layer (68 nm), bis(10-hydroxybenzo[h]quinolinato)-beryllium (Bebq_2_) as a light-emitting layer (host), and tris[1-phenylisoquinoline-C2,N]iridium(III) (Ir(piq)_3_) as a light-emitting layer (guest, 70 nm, 8 wt%). The green OLED has 4,4′-cyclohexylidenebis[N,N-bis(4-methylphenyl)benzenamine] (TAPC) as a hole transport layer (40 nm), tris(4-carbazoyl-9-ylphenyl)amine (TCTA) as a light-emitting layer (host), (tris[2-phenylpyridinato-C2,N]iridium(III)) (Ir(ppy)_3_) as a light-emitting layer (guest, 25 nm, 8 wt%), and 4,6-bis(3,5-di(pyridin-3-yl)phenyl)-2-methylpyrimidine (B3PYMPM) as an electron transport layer (35 nm). The blue OLED has NPB as a hole transport layer (45 nm), 2-methyl-9,10-bis(naphthalen-2-yl)anthracene (MADN) as a light-emitting layer (host), p-bis(p-N,N-diphenyl-aminostyryl) benzene (DSA-Ph) as a light-emitting layer (guest, 25 nm 3 wt%), and tris(8-hydroxyquinolinato)aluminium (Alq_3_) as an electron transport layer (10 nm).

### Barrier fabrication

The ZAM film was deposited using ALD equipment with H_2_O as a reactant. The ALD process was conducted at a chamber temperature of 70 °C and a vacuum of 3.0 × 10^−1^ Torr. ZnO was fabricated using a dimethylzinc source, Al_2_O_3_ was fabricated using a trimethylaluminium source, and MgO was fabricated using a bis-ethyl-cyclopentadienyl-magnesium source. ZnO, Al_2_O_3_, and MgO were grown at growth rates of 1.03 Å/cycle, 0.83 Å/cycle and 1.00 Å/cycle, respectively. The Si-based polymer (i-OPTO BC 10, INTECH Nano Materials) was fabricated by spin coating. The spin coating was accelerated for 30 s to 5000 rpm and held at this speed for 3 s. After spin coating, a curing process was carried out in a 70 °C ALD chamber for 20 min.

### Device characterization

The electrical properties of the OLED were measured using a source meter (Keithley 2400, Keithley Inc.), and the optical properties were measured using a spectroradiometer (CS-2000, Konica Minolta Inc.). The operating life reliability of the OLED was measured under a constant current drive in an air environment using a Si photodiode (Polaronix M9000S, McScience Inc.). In addition, the cross-sectional structure and thickness of the OLEDs were measured by FIB-SEM equipment (Helios Nanolab 450, FEI Inc.). Barrier WVTR values were measured by the conductance change in a calcium pad in the calcium test (Keithley 2750, Keithley Inc.). These calcium tests were carried out at 30 °C and 90% humidity. The barrier transmittance was measured with a UV-visible spectrometer (UV-2550, Shimadzu Inc.), and the surface roughness was measured with an atomic force microscope (XE-100, Park Systems Inc.). The flexibility of the textiles was measured with cantilever equipment (Cantilever tester, Mcscience Inc.), and the adhesion was measured using a peeling tester (SMA-500N). The neutral axis was measured by mechanical simulation (ANSYS), and the amount of light per distance was measured by ray tracing simulations (LightTools).

### In vitro 2D cell-based experiments (cultured normal human keratinocytes)

#### Cell culture

All samples were obtained with informed consent, and fibroblasts and keratinocytes were separated from the foreskin of humans obtained by circumcision. Cell cultures were carried out in accordance with relevant guidelines and regulations. Cell culture was performed according to the guidelines approved by the Institutional Review Board (IRB Approval No. B-1603/340-309) of Seoul National University’s Bundang Hospital. We treated skin specimens according to the ref. ^62^ and modified them using thermolysin^21^. Fibroblasts were cultured according to the method described in a previous paper^21^. Keratinocytes were cultured in keratinocyte growth medium (KGM, CC-3111, Lonza, Walkersville, MD).

#### Cell cytotoxicity and cell proliferation test based on the cell metabolic activity

The cytotoxicity and proliferation effect of the STOLEDs on keratinocytes was tested by Cell Counting Kit-8 (CCK-8, Dojindo, Kumamoto, Japan) assays. The keratinocytes were seeded into 96-well plates at 8000 or 4000 cells/well for the cytotoxicity test or cell proliferation test, respectively, and cultured (24 h). Subsequently, the cells were serum-starved for 6 h and then irradiated with a STOLED according to the indicated conditions. CCK-8 solution was added to each well for 24 h for the cytotoxicity test or 72 h for the proliferation test and incubated for an additional 2 h. The absorbance at 450 nm was measured using a SPECTRAmax Plus 384 microplate spectrophotometer (Molecular Devices, Sunnyvale, Calif.).

#### Migration test (wound healing)

Keratinocytes were seeded into 96-well ImageLock microplates (4379, Essen BioScience, Ann Arbor, MI) at 35,000 cells/well and cultured (24 h). Thereafter, the cells were serum-starved for 6 h, and then, the confluent cell layer was scratched by a 96-well wound-maker (Essen BioScience) to make wounds according to the instructions and then irradiated with a STOLED according to the indicated conditions. Photographs were obtained using an IncheCyte ZOOM Imaging System (Essen BioScience) at 3-h intervals for 30 h after STOLED irradiation. The relative wound density was measured by quantifying cell migration using recorded images of the initial wound and wound closure.

#### Semi-in-vivo 3D artificial skin model experiments

Skin models were prepared and maintained using our method^63^. They were irradiated with OLEDs on the second day of incubation at the air-liquid interface and cultured for an additional 10 days. They were fixed in 10% formaldehyde for 1 day and processed for conventional paraffin embedment. For histological analysis, Masson’s trichrome staining was performed. Images were obtained using a light microscope system (BX53, Olympus, Tokyo, Japan).

#### Organ culture experiments 1

All protocols involving rats were approved by the Institutional Animal Care and Use Committee of Seoul National University Bundang Hospital. Approximately sixteen millimetre full-thickness skin biopsies from the backs of 7-week-old Sprague Dawley rats were obtained. They were irradiated with OLEDs, and a 6 mm hole was cut out in the middle to make a wound. Then, they were incubated for 3 weeks in DMEM supplemented with 10% foetal bovine serum (FBS, Thermo Scientific HyClone, Logan, UT). They were photographed, and the images were analysed.

#### Organ culture experiments 2

All protocols involving rats were approved by the Institutional Animal Care and Use Committee of Seoul National University Bundang Hospital. A one centimetre diameter rod cooled by liquid nitrogen was applied to the back of a 7-week-old Sprague Dawley rat for 10 s. When the colour of the skin had recovered, the rod was applied for another 5 s to make a cryo-wound. A day later, the dead epidermal cells were peeled off. Approximately 25-millimeter full-thickness skin biopsies, including the wound in the middle, were obtained. They were irradiated with OLEDs and then incubated for 8 days in DMEM supplemented with 10% FBS. They were fixed in 10% formaldehyde for 1 day and processed for conventional paraffin embedment. For histological analysis, sections were stained with haematoxylin and eosin (H&E). Images were obtained with a light microscope system (BX53) and analysed by software (analysis TS Lite, Olympus). For cell nucleus analysis, sections were de-paraffinized and stained with 4’,6-diamidine-2’-phenylindole dihydrochloride (DAPI, 10236276001, Roche, Mannheim, Germany). Fluorescent images were obtained with a microscope (Axio Imager A1, Carl Zeiss, Jena, Germany) and analysed by AxioVision software (Carl Zeiss).

## Supplementary information

Supplementary Information for Sandwich-structure Transferable Free-form OLEDs for Wearable and Disposable Skin Wound Photomedicine

Supplementary Video S1

Supplementary Video S2

Supplementary Video S3

Supplementary Video S4
