# Supplementary material for: Sandwich-structure transferable free-form OLEDs for wearable and disposable skin wound photomedicine
Source: Light Sci Appl. 2019 Dec 9;8:114. doi: 10.1038/s41377-019-0221-3 (PMC6900403; doi:10.1038/s41377-019-0221-3)
Supplement: Supplementary file 1 — Supplementary Information for Sandwich-structure Transferable Free-form OLEDs for Wearable and Disposable Skin Wound Photomedicine [file 41377_2019_221_MOESM1_ESM.docx]

**Supplementary Information**

**Sandwich-structure Transferable Free-form OLEDs for Wearable and Disposable Skin Wound Photomedicine**

Yongmin Jeon^1†^, Hye-Ryung Choi^2†^,Jeong Hyun Kwon^1^, Seongyeop Choi^1^, Kyung Mi Nam^2^ , Kyoung-Chan Park^2*^ and Kyung Cheol Choi^1^^*^

^1^School of Electrical Engineering, Korea Advanced Institute of Science and Technology (KAIST), Daejeon 34141, Republic of Korea.

^2^Department of Dermatology, Seoul National University Bundang Hospital (SNUBH), Seongnam 13620, Republic of Korea

^†^These authors contributed equally to this work.

^*^Corresponding author : [kyungcc@kaist.ac.kr](mailto:kyungcc@kaist.ac.kr) (Kyung Cheol Choi), [gcpark@snu.ac.kr](mailto:gcpark@snu.ac.kr) (Kyoung-Chan Park).

**Supplementary Figures**


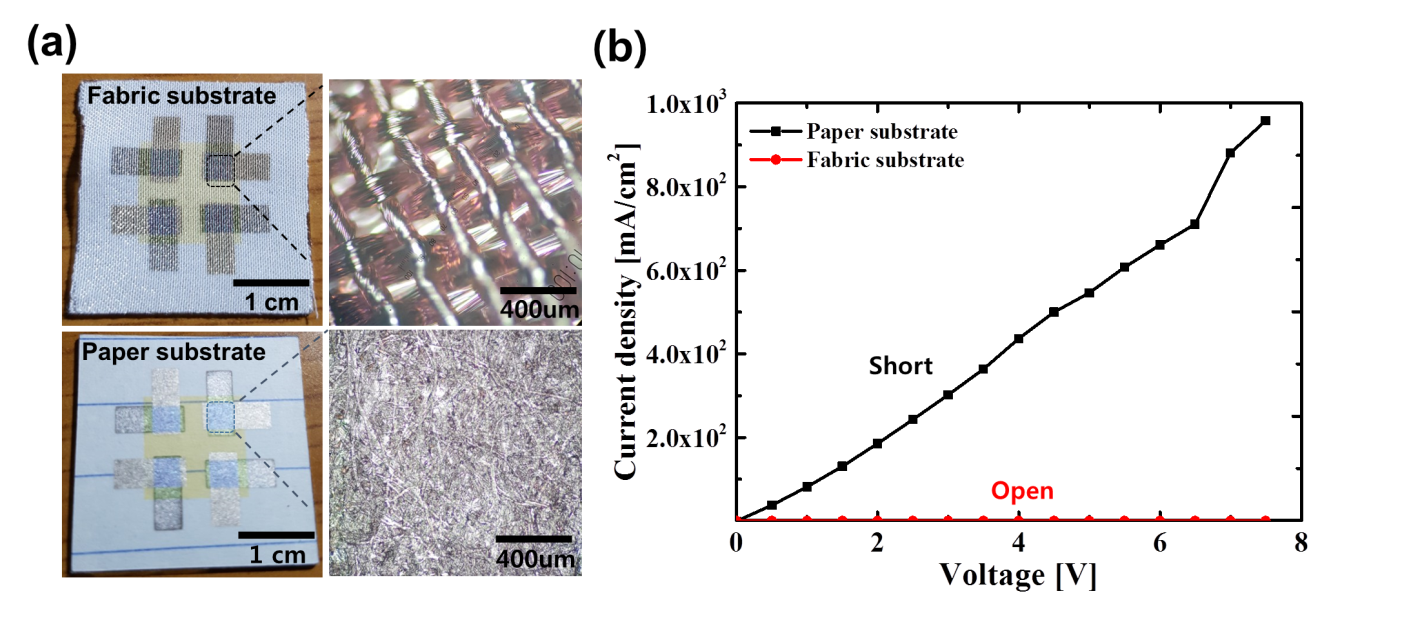
**Figure S1. A typical OLED fabricated on a substrate that is not planarized**: **(a)** Photographs of OLED devices fabricated directly on paper and textile with high roughness. **(b)** J / V electrical characteristics of each fabricated device.


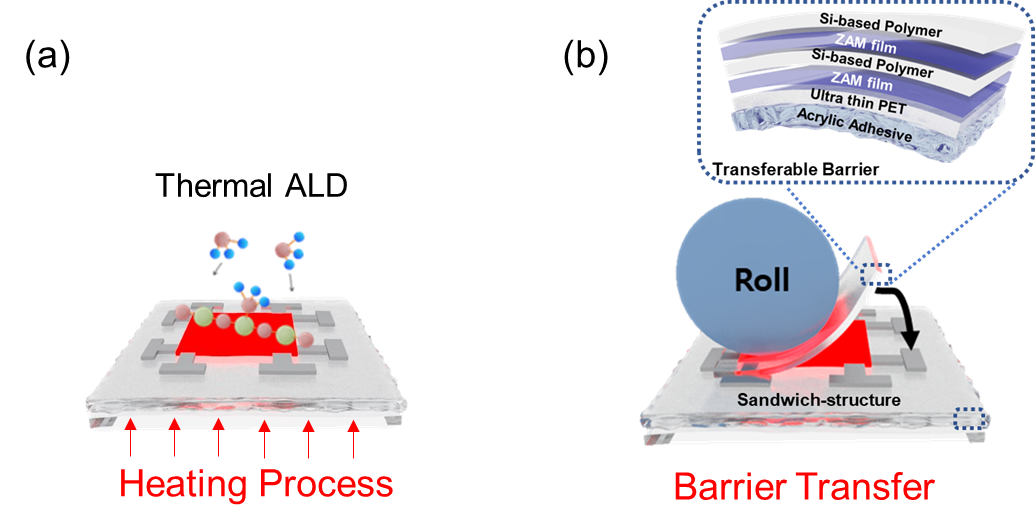


**Figure S2. Comparison of barrier formation method for Unusual OLEDs** (a) Barrier formation method using thermal ALD (b) Transfer method for the barrier in this study.


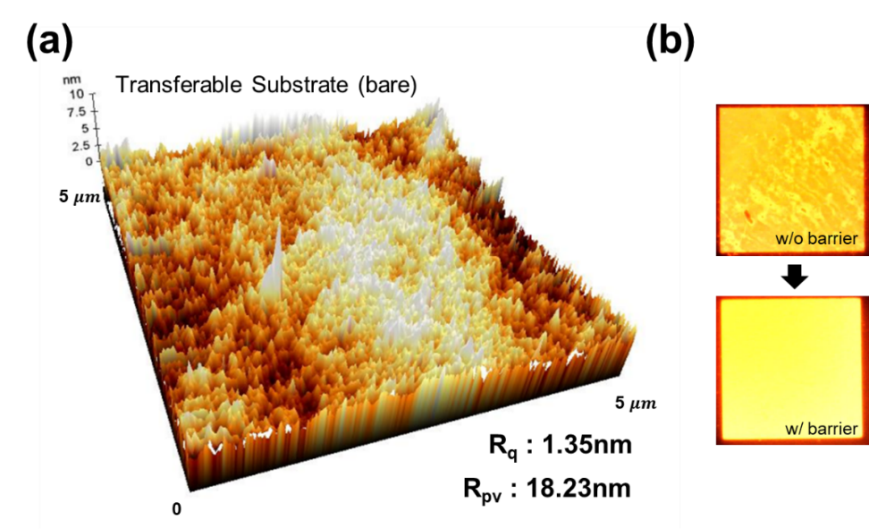


**Figure S3. Properties of transferable substrates without barrier formation: (a)** AFM image and surface roughness. **(b)** Picture of OLED with and without barrier.


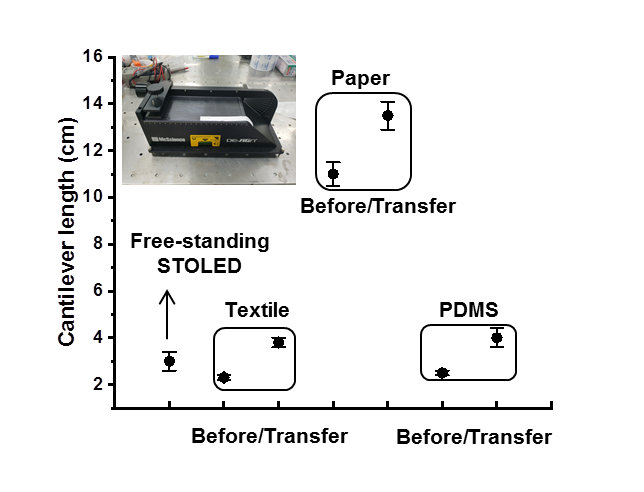


**Figure S4. Flexibility evaluation graph of the Peirce cantilever test for various substrates**


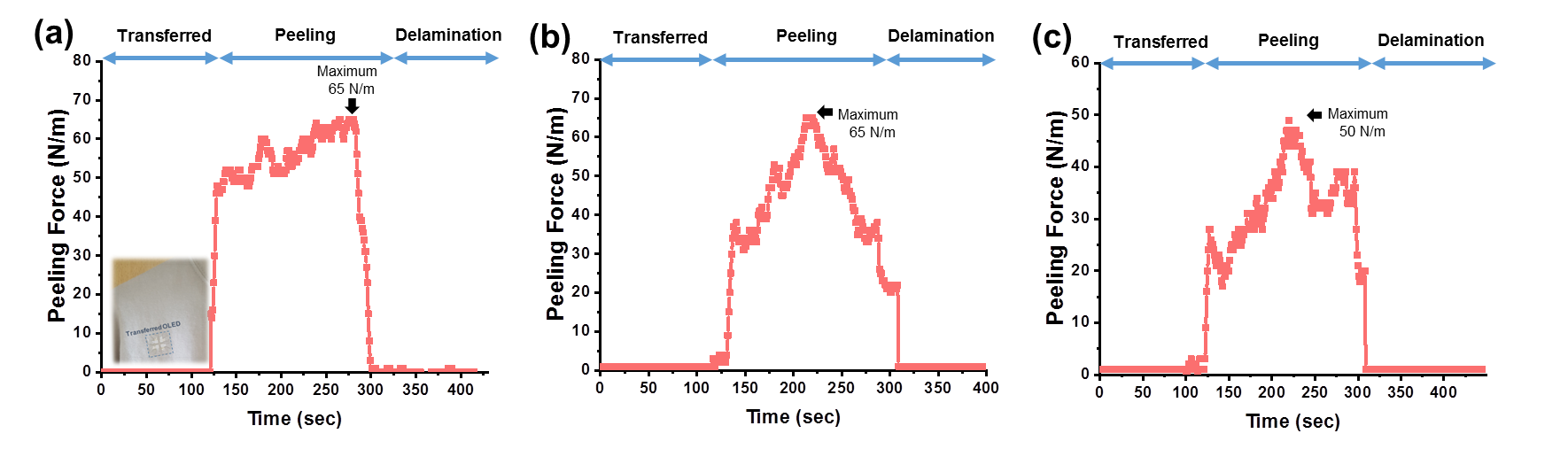


**Figure S5. Peeling test to evaluate adhesion force : (a)** STOLED transferred to textile substrate **(b)** STOLED transferred to paper substrate **(c)** STOLED transferred to PDMS substrate


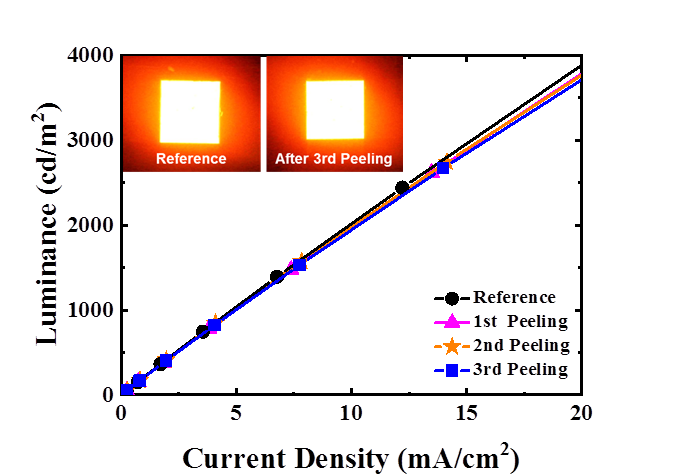


**Figure S6. Lighting performance test of STOLED after peeling from textile substrate**


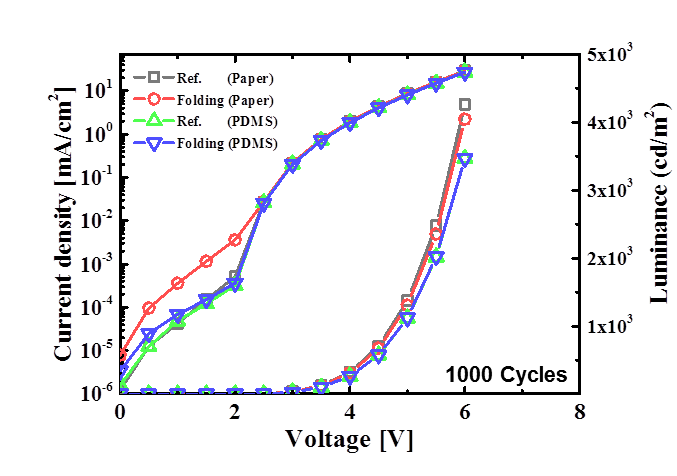


**Figure S7. STOLED folding test on PDMS (R = 350**$\boldsymbol{\mu m}$**) and paper substrate (R = 500** $\boldsymbol{\mu m}$**)**


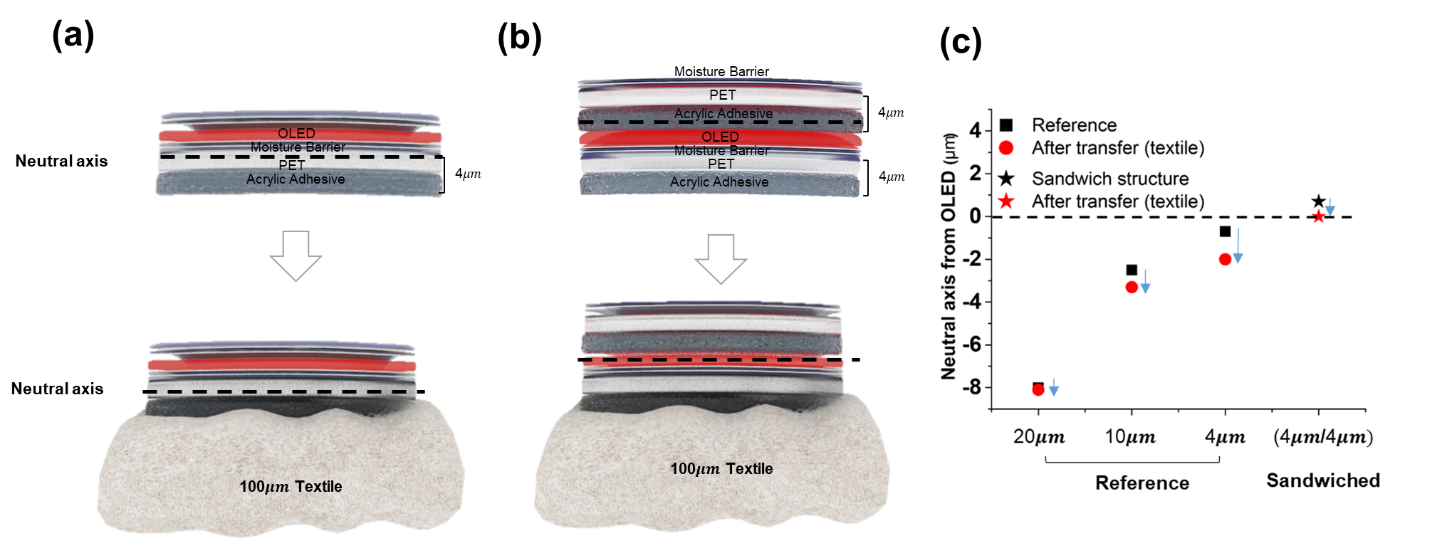


**Figure S8. Neutral axis analysis of free-form OLEDs transferred to textile: (a)** Structure of general reference STOLED and change in neutral axis before and after transfer. **(b)** STOLED sandwich structure and change in neutral axis before and after transfer. **(c)** A graph showing the distance of the neutral axis from the center of the OLED, depending on the structure.


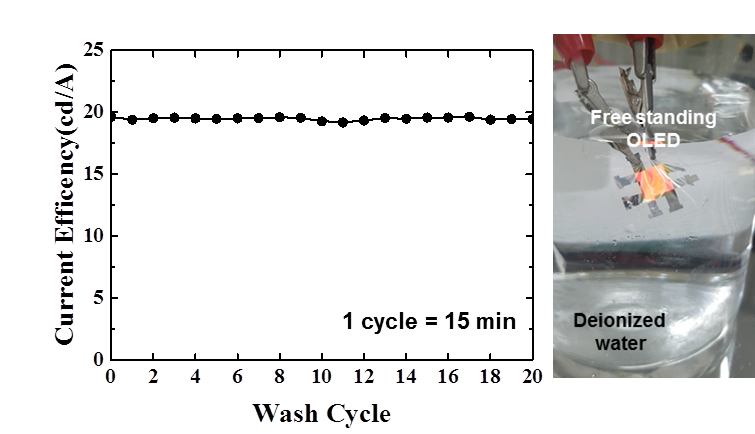


**Figure S9. Current efficiency characteristic after washing test of device.**


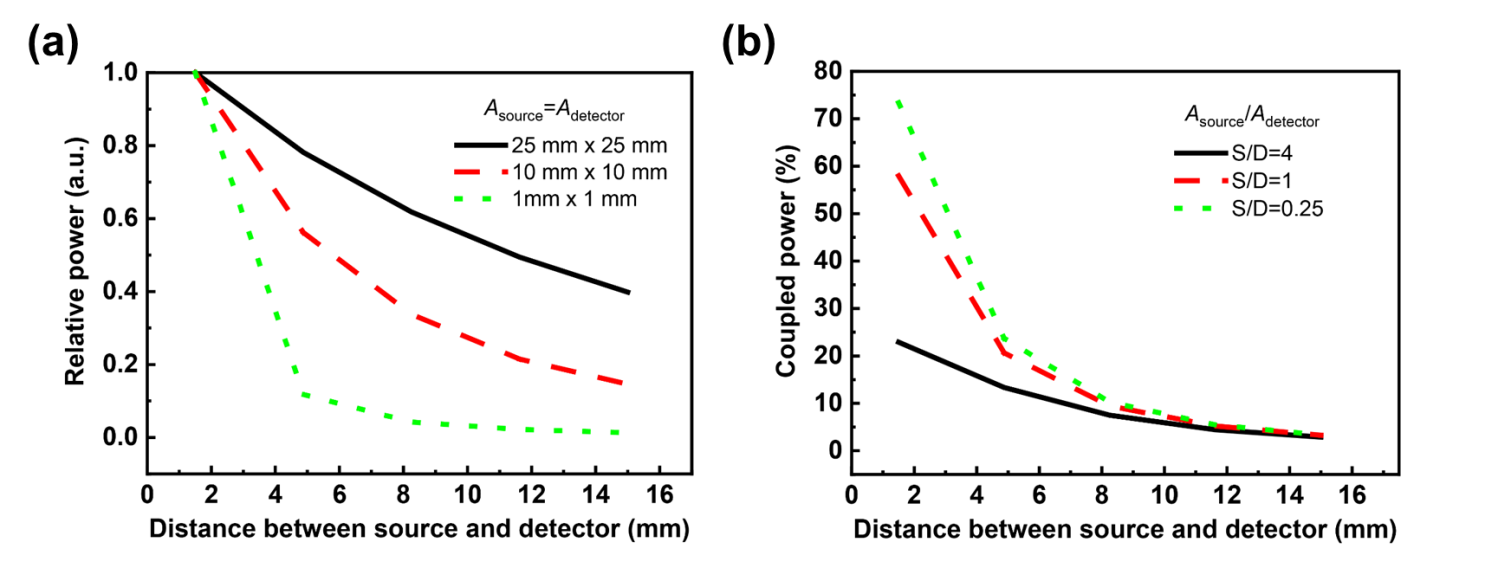


**Figure S10. The ratio of light intensity according to the distance between the light source and the detector: (a)** When the light source and detector are the same size. **(b)** When the light source and detector sizes are different.


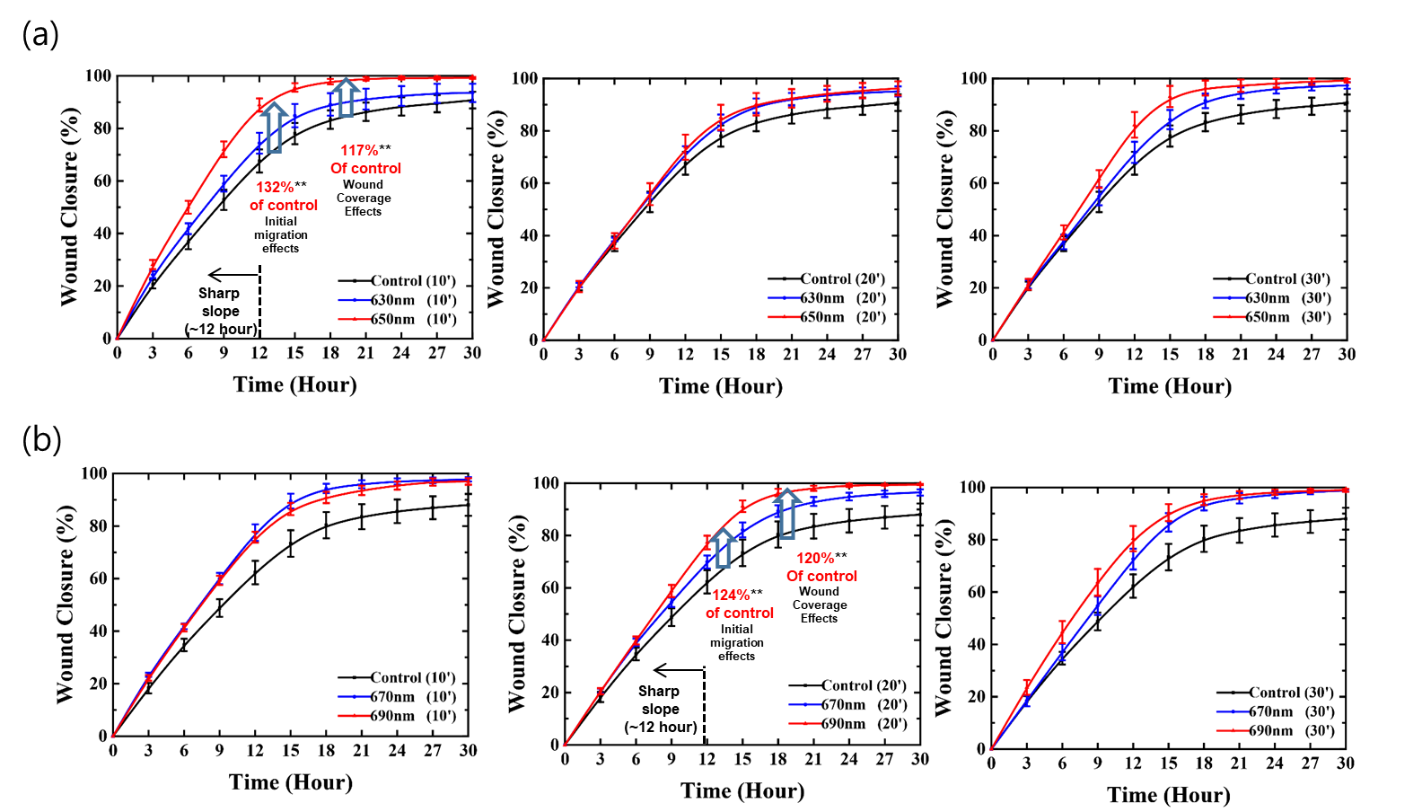


**Figure S11. Cell migration test of keratinocytes according to wavelength and energy after red STOLED irradiation. (The graphs are expressed as mean ± mean error (n = 8), and all STOLEDs with peak wavelengths between 630 and 690 nm were irradiated with a power of 5 mW cm^-2^. Also,** *** means that the p value is less than 0.05 and ** means that the p value is less than 0.01): (a)** The graph of keratinocytes cell migration when irradiated for 10, 20 and 30 minutes by STOLED with 630 nm and 650 nm peak wavelengths, respectively. **(b)** The graph of keratinocytes cell migration when irradiated for 10, 20 and 30 minutes by STOLED with 670 nm and 690 nm peak wavelengths, respectively.


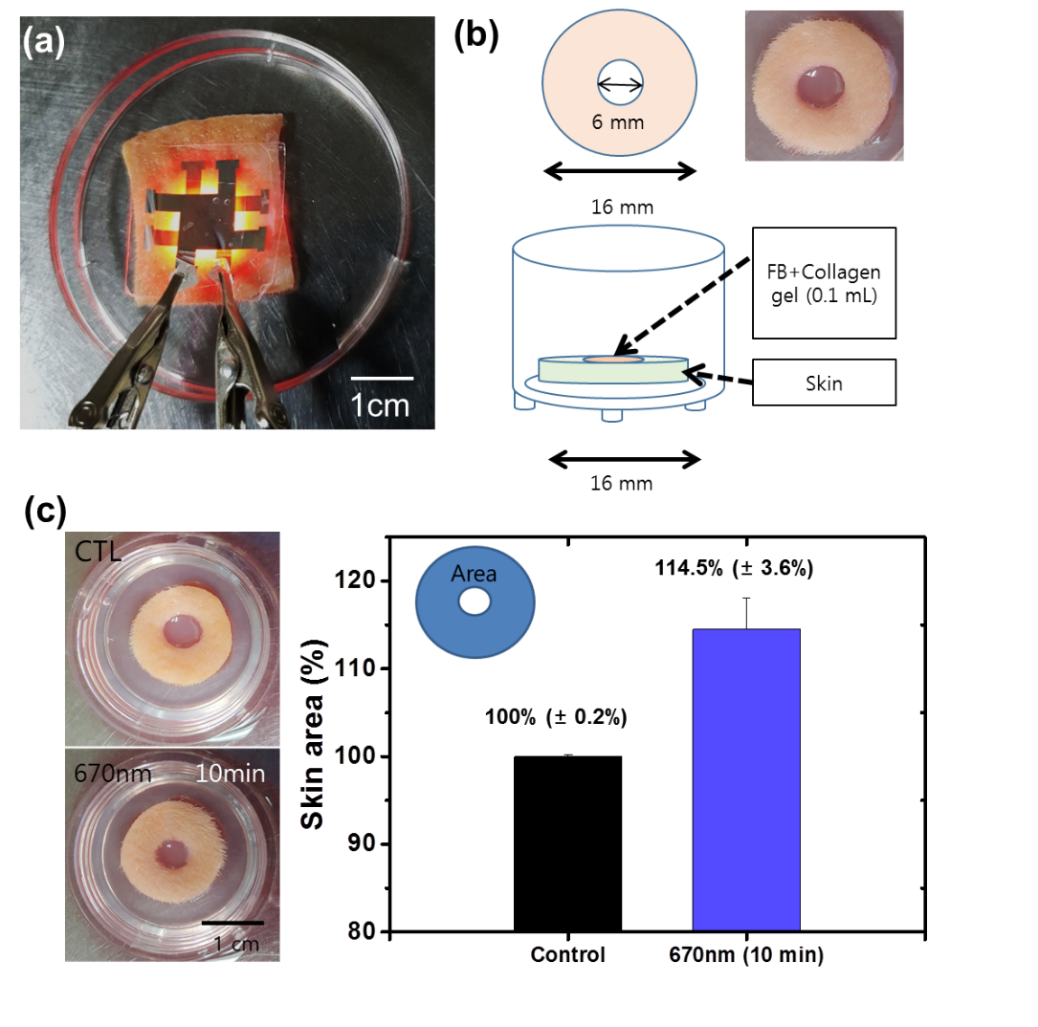


**Figure S12. The effect on organ culture after irradiating skin fragments of rats with STOLED: (a)** The photograph shows the STOLED irradiating the skin of a rat with a 6 mm diameter hole. **(b)** Organ culture condition of rat skin after light irradiation. **(c)** Graph of rat skin area 3 weeks after irradiation by STOLED.

**Supplementary Videos**

**Supplementary Video S1**

A demonstration video of a textile-based free-form OLED.

**Supplementary Video S2**

A demonstration video of a paper-based free-form OLED.

**Supplementary Video S3**

A demonstration video of a rubber skin and cylinder-based free-form OLED.

**Supplementary Video S4**

**A demonstration video where textile-based OLEDs are driven under folding conditions.**

**Supplementary Tables**

**Table S1. Comparison of key performance and reliability values of previously reported unusual optoelectronic devices with the STOLED in this study**

| Type | Substrate | Thickness | Efficiency | Flexibility | Barrier | Ref. |
| --- | --- | --- | --- | --- | --- | --- |
| This work  (STOLED) | Free-form  (Transferable) | 10 $\mu m$ | 79.4 cd A^-1^ | 350$\mu m$ | Free-form  (Transferable) | **Ours** |
| Textile-OLED | PEN-based Textile | 40-50 $\mu m$ | 49.1 cd A^-1^ | 2000 $\mu m$ | Thermal ALD | **^1^** |
| Textile-OLED | Polyester Textile | 40-50 $\mu m$ | 5.5 cd A^-1^ | 20000 $\mu m$ | Thermal ALD | **^2^** |
| Textile-OLED | Polyester Textile | 40-50 $\mu m$ | 9.0 cd A^-1^ | 5000 $\mu m$ | - | **^3^** |
| Textile-ELD | PET mesh Textile | 150 $\mu m$ | - | - | - | **^4^** |
| Paper-OLED | Cellulose Paper | 40 $\mu m$ | 47.0 cd A^-1^ | - | - | **^5^** |
| Paper-OLED | Chitin Paper | 40 $\mu m$ | 5.1 cd A^-1^ | - | - | **^6^** |
| Paper-OLED | Cellulose Paper | 300 $\mu m$ | < 1 cd A^-1^ | - | - | **^7^** |
| Paper-OLED | Cellulose Paper | - | 53.7 cd A^-1^ | - | - | **^8^** |
| Paper-OLED | Cellulose Paper | 25 $\mu m$ | 32.0 cd A^-1^ | - | - | **^9^** |
| Fiber-PLEC | Stainless-steel Fiber | 510 $\mu m$ | 0.8 cd A^-1^ | 6000 $\mu m$ | - | **^10^** |
| Fiber-PLED | PET-based Fiber | 90$\mu m$ | 11.6 cd A^-1^ | 3000 $\mu m$ | Thermal ALD | **^11^** |
| Fiber-PLED | PET-based Fiber | 300$\mu m$ | 3.0 cd A^-1^ | 2500 $\mu m$ | Thermal ALD | **^12^** |
| Fiber-OLED | Silica Fiber | 480 $\mu m$ | < 1 cd A^-1^ | - | - | **^13^** |
| Fiber-OLED | Glass Fiber | 250$\mu m$ | 10.0 cd A^-1^ | - | - | **^14^** |

**Supplementary References**

1 Choi, S. *et al.* Highly flexible and efficient fabric-based organic light-emitting devices for clothing-shaped wearable displays. *Scientific Reports* **7**, 6424 (2017).

2 Kim, W. *et al.* Reliable actual fabric‐based organic light‐emitting diodes: toward a wearable display. *Advanced Electronic Materials* **2**, 1600220 (2016).

3 Kim, W. *et al.* Soft fabric-based flexible organic light-emitting diodes. *Organic Electronics* **14**, 3007-3013 (2013).

4 Hu, B. *et al.* Textile‐Based Flexible Electroluminescent Devices. *Advanced Functional Materials* **21**, 305-311 (2011).

5 Purandare, S., Gomez, E. F. & Steckl, A. J. J. N. High brightness phosphorescent organic light emitting diodes on transparent and flexible cellulose films. *Nanotechnology* **25**, 094012 (2014).

6 Jin, J. *et al.* Chitin nanofiber transparent paper for flexible green electronics. *Advanced Materials* **28**, 5169-5175 (2016).

7 Ummartyotin, S., Juntaro, J., Sain, M., Manuspiya, H. J. I. C. & Products. Development of transparent bacterial cellulose nanocomposite film as substrate for flexible organic light emitting diode (OLED) display. *Industrial Crops and Products* **35**, 92-97 (2012).

8 Najafabadi, E., Zhou, Y., Knauer, K., Fuentes-Hernandez, C. & Kippelen, B. J. A. P. L. Efficient organic light-emitting diodes fabricated on cellulose nanocrystal substrates. *Applied Physics Letters* **105**, 124_121 (2014).

9 Gomez, E. F. & Steckl, A. J. J. A. P. Improved performance of OLEDs on cellulose/epoxy substrate using adenine as a hole injection layer. *ACS Photonics* **2**, 439-445 (2015).

10 Zhang, Z. *et al.* A colour-tunable, weavable fibre-shaped polymer light-emitting electrochemical cell. *Nature Photonics* **9**, 233 (2015).

11 Kwon, S. *et al.* Weavable and highly efficient organic light-emitting fibers for wearable electronics: a scalable, low-temperature process. *Nano letters* **18**, 347-356 (2017).

12 Kwon, S. *et al.* High Luminance Fiber‐Based Polymer Light‐Emitting Devices by a Dip‐Coating Method. *Advanced Electronic Materials* **1**, 1500103 (2015).

13 O'Connor, B., An, K. H., Zhao, Y., Pipe, K. P. & Shtein, M. J. A. M. Fiber shaped light emitting device. *Advanced Materials* **19**, 3897-3900 (2007).

14 Ko, K.-J. *et al.* High-performance, color-tunable fiber shaped organic light-emitting diodes. *Nanoscale* **10**, 16184-16192 (2018).
